# Light gradient boost tree classifier predictions on appendicitis with periodontal disease from biochemical and clinical parameters

**DOI:** 10.3389/froh.2024.1462873

**Published:** 2024-09-13

**Authors:** Pradeep Kumar Yadalam, Prathiksha Vedhavalli Thirukkumaran, Prabhu Manickam Natarajan, Carlos M. Ardila

**Affiliations:** ^1^ Department of Periodontics, Saveetha Dental College, SIMATS, Saveetha University, Chennai, India; ^2^ Saveetha Institute of Medical and Technical Science [SIMATS], Saveetha University, Chennai, India; ^3^ Department of Clinical Sciences, Center of Medical and Bio-Allied Health Sciences and Research, College of Dentistry, Ajman University, Ajman, United Arab Emirates; ^4^ Department of Basic Sciences, Universidad de Antioquia U de A, Medellín, Colombia; ^5^ Biomedical Stomatology Research Group, Universidad de Antioquia U de A, Medellín, Colombia

**Keywords:** periodontitis, systemic diseases, appendicitis, machine learning, artificial intelligence

## Abstract

**Introduction:**

Untreated periodontitis significantly increases the risk of tooth loss, often delaying treatment due to asymptomatic phases. Recent studies have increasingly associated poor dental health with conditions such as rheumatoid arthritis, diabetes, obesity, pneumonia, cardiovascular disease, and renal illness. Despite these connections, limited research has investigated the relationship between appendicitis and periodontal disease. This study aims to predict appendicitis in patients with periodontal disease using biochemical and clinical parameters through the application of a light gradient boost tree classifier.

**Methods:**

Data from 125 patient records at Saveetha Institute of Dental College and Medical College were pre-processed and analyzed. We utilized data preprocessing techniques, feature selection methods, and model development approaches to estimate the risk of appendicitis in patients with periodontitis. Both Random Forest and Light Gradient Boosting algorithms were evaluated for accuracy using confusion matrices to assess their predictive performance.

**Results:**

The Random Forest model achieved an accuracy of 94%, demonstrating robust predictive capability in this context. In contrast, the Light Gradient Boost algorithms achieved a significantly higher accuracy of 98%, underscoring their superior predictive efficiency. This substantial difference highlights the importance of algorithm selection and optimization in developing reliable predictive models. The higher accuracy of Light Gradient Boost algorithms suggests effective minimization of prediction errors and improved differentiation between appendicitis with periodontitis and healthy states. Our study identifies age, white blood cell count, and symptom duration as pivotal predictors for detecting concurrent periodontitis in acute appendicitis cases.

**Conclusions:**

The newly developed prediction model introduces a novel and promising approach, providing valuable insights into distinguishing between periodontitis and acute appendicitis. These findings highlight the potential to improve diagnostic accuracy and support informed clinical decision-making in patients presenting with both conditions, offering new avenues for optimizing patient care strategies.

## Introduction

Acute appendicitis, a prevalent and potentially life-threatening condition, involves the inflammation of the appendix, a small, finger-like organ attached to the large intestine. Although the exact function of the appendix remains unclear, blockages—often due to fecaliths or hardened fecal matter—can lead to infection and inflammation (1, 2). The initial symptom typically involves abdominal pain that begins near the navel and migrates to the lower right abdomen. Other symptoms may include fever, vomiting, nausea, and loss of appetite. Prompt diagnosis and surgical removal of the inflamed appendix, known as an appendectomy, are crucial to preventing complications such as abscess formation, perforation, and peritonitis (2, 3).

The precise etiology of acute appendicitis is still not fully understood; however, possible causes include tumors, parasites, lymphoid hyperplasia, and fecaliths. Timely intervention is essential to minimize the risk of complications and ensure a positive outcome for those suffering from acute appendicitis. Acute appendicitis (AA) is a common cause of acute abdominal pain, particularly in individuals aged 10–30. Delayed diagnosis can lead to severe complications, including abscess, plastron, perforation, or peritonitis, which can be fatal in complex cases. Approximately 7% of the population experiences delays in diagnosis (4, 5). While the exact cause remains uncertain, common contributors include tumors, lymphoid hyperplasia, fecaliths, and parasites. Notably, appendicitis involves a polymicrobial process with both aerobic and anaerobic bacteria in acute and complex cases. Identifying these causes promptly is imperative to mitigate the adverse effects of acute appendicitis (6, 7).

Research into the connection between oral infections and systemic illnesses has surged since Hunter's 1900 introduction of focal disease theory (8, 9). A 1989 study in Finland linked heart disease with oral illnesses, spurring further investigation into causal mechanisms, biological plausibility, and associations. Periodontal diseases, which are infectious and inflammatory conditions of the tissues surrounding the teeth, have both local and systemic effects. The estimated area of untreated periodontal inflammation, indicated by bleeding and probing depth, ranges from 15 to 72 cm² and is associated with systemic inflammation markers such as C-reactive protein (10, 11).

The common risk factor approach highlights the importance of managing shared risk factors to effectively treat various diseases. Mechanisms connecting periodontal diseases to systemic problems include immune responses, hematogenous dissemination of immune/infection components, and bacteremia (12, 13). Research shows that bacteremia can occur through chewing and brushing, and signs of systemic inflammation may diminish following periodontal therapy. These findings emphasize the intricate relationship between oral health and overall health. Aspects of oral health include the absence of periodontal disease, dental problems, and psychosocial concerns about oral functions. Recent studies have linked poor dental health to systemic diseases such as rheumatoid arthritis, diabetes, obesity, pneumonia, cardiovascular disease, and renal diseases (14).

Oral microorganisms and their byproducts can contribute to infections in various body regions through mechanisms such as metastatic infection, damage from microbial toxins, and inflammation in immunocompromised individuals. Studies by Blod et al. and Aiyoshi et al. have detected oral bacterial pathogens, including Fusobacterium species, in the appendix, suggesting that the oral cavity may serve as a reservoir for AA (6, 15). Notably, *Parvimonas*, a bacterium associated with periodontal disease, is prevalent among the pathogens isolated from AA patients. The presence of *Fusobacterium* and *Parvimonas* in the appendix may promote biofilm formation, thereby contributing to mucosal inflammation and the pathogenesis of AA.

In oral healthcare, machine learning-based illness prediction using clinical data parameters holds significant promise. Advanced algorithms, such as light gradient boosting trees, analyze and interpret large patient datasets to improve forecasts, early detection, and personalized treatment strategies. These algorithms utilize clinical factors such as patient demographics, medical history, test results, and imaging findings. By training a machine learning model on a dataset with known outcomes, the algorithm learns patterns and relationships within the data. The Light Gradient Boosting Tree [LightGBM] (16–18) enhances gradient boosting by improving training efficiency and prediction accuracy in large datasets. The ensemble machine learning technique Random Forest, which combines multiple decision trees, also improves forecast accuracy and manages complex datasets. Few studies have focused on predicting acute appendicitis and periodontal disease using advanced machine learning techniques. This study aims to predict periodontal disease-related appendicitis using Light Gradient Boost Tree classifiers based on clinical parameters, highlighting a novel approach that integrates oral health data with systemic disease prediction to improve patient outcomes.

## Materials and methods

This study was approved by the Ethical Committee of Saveetha Medical College and Hospital (Ethical approval code IHEC/346). Informed and ethical consent was not required as 125 data points were gathered from previous records at the Saveetha Institute of Dental College and Medical College. The procedures included data collection, preprocessing, model selection, training, evaluation, hyperparameter tuning, cross-validation, and making the data interpretable using the DataRobot tool (https://app.datarobot.com). Preprocessing and exploratory analysis were performed on the data. The recorded clinical data and biochemical parameters included platelet count, neutrophils, basophils, eosinophils, total leukocytes, total neutrophils, monocytes, lymphocytes, and periodontal clinical parameters. The data were classified into acute appendicitis, appendicitis with periodontal disease, and normal categories.

Light Gradient Boosted Trees (LightGBM) (1, 19) stands out as a robust machine learning algorithm renowned for its efficiency and speed, particularly well-suited for processing large-scale datasets and high-dimensional feature spaces. Our study leverages LightGBM to explore its application in predicting appendicitis among patients with periodontal disease.

LightGBM operates within the gradient boosting framework, which involves sequentially building an ensemble of weak learners, typically decision trees. Each subsequent model in the ensemble corrects errors made by its predecessors, thereby improving overall predictive accuracy.

Characteristics of LightGBM:

1. Gradient Boosting Framework: The gradient boosting framework, on which LightGBM is based, constructs an ensemble of weak learners (often decision trees) sequentially. Each tree improves overall predictive performance by correcting the errors of the previous one.
2. LightGBM's Distinctive Features: LightGBM introduces several elements that enhance its efficiency. Notably, it constructs trees using a histogram-based learning strategy. This approach reduces computation time by creating histograms of feature values and selecting the best split points from them, rather than using all data points to find the optimal split.
3. Leaf-Wise Tree Growth: Unlike traditional gradient boosting, which uses a level-wise approach, LightGBM employs a leaf-wise tree growth strategy. This method expands the leaf with the greatest delta loss, creating a more complex and potentially deeper tree structure capable of capturing intricate patterns in the data.
4. Gradient-Based One-Side Sampling (GOSS): Gradient-based one-sided sampling is a technique used by LightGBM that focuses on training cases with larger gradients. By eliminating less informative samples, GOSS shortens the training time and reduces the overall computational load.
5. Categorical Feature Support: LightGBM effectively handles categorical features without requiring one-hot encoding, simplifying preprocessing procedures and preserving important information.
6. Regularization and Shrinkage: LightGBM includes regularization terms and shrinkage to reduce overfitting and improve the model's generalization to new data.

Random Forest (20) is a widely used ensemble learning technique known for its robustness in handling both regression and classification tasks. In our study, we explore the application of Random Forest in predicting appendicitis in patients with periodontal disease, leveraging its capabilities in ensemble learning.

Random Forest constructs multiple decision trees during the training process, each tree contributing to the final prediction through a combination mechanism that ensures robust performance across various datasets.

Characteristics of Random Forest:

1. Bootstrap Sampling [Bagging]: Bootstrap sampling generates random portions of the training data so that each tree can be trained on a slightly different dataset.
2. Feature Randomization: A random subset of features is considered for splitting at each decision tree node. This process increases tree diversity and helps avoid overfitting.
3. Tree Construction: Decision trees are constructed by recursively dividing the data into subsets based on the most discriminative features using the selected features.
4. Voting or Averaging: For classification, the final prediction is determined by a majority vote among the trees, whereas for regression, the final forecast is the average of each tree's prediction.
5. Ensemble Output: The outputs of each decision tree are combined to produce a reliable, accurate model that performs well on new data.

## Results

The analysis of the biochemical data and the performance of various machine learning classifiers in predicting appendicitis with periodontitis yielded significant findings.

**Figure 1:**
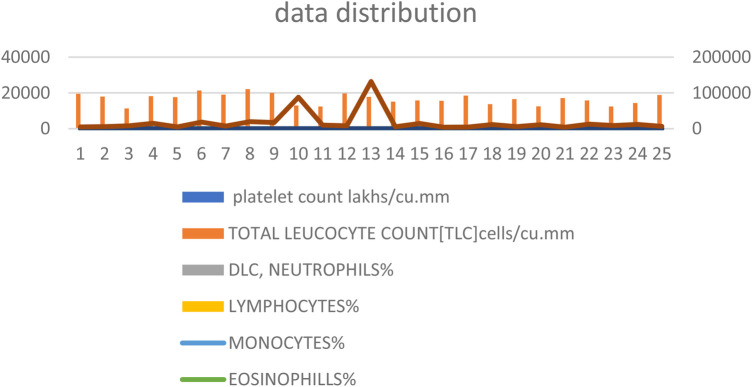
Distribution of biochemical data. This figure shows the spread and central tendencies for key biochemical parameters including platelet count, neutrophils, lymphocytes, monocytes, and eosinophils. The distribution is visualized to highlight the variability and central values within the dataset, providing a clear overview of the data's overall range and distribution patterns.

Figure 1 presents the distribution of the biochemical data, highlighting the variability and central tendencies within the dataset.

Table 1 summarizes the mean values and standard deviations (SD) for key biochemical parameters: platelet count (measured in lakhs/cu.mm), percentage of neutrophils (DLC, neutrophils%), lymphocytes%, monocytes%, and eosinophils%.

**Table 1:** Mean and SD of biochemical parameters.

Parameter	Platelet count lakhs/cu.mm	DLC, neutrophils%	Lymphocytes%	Monocytes%	Eosinophills%
Mean	2.67135	76.79218966	22.74135345	4.381465517	2.775896552
SD	0.95386	66.61351116	12.89328715	1.958166918	3.081135682

The mean values represent the average levels of these parameters within the dataset, providing insights into their central tendencies. The standard deviations quantify the variability or dispersion of data points around the mean, indicating the degree of fluctuation in these biochemical measurements. These statistical measures are essential for characterizing the distribution and stability of the biochemical profile studied, crucial for interpreting clinical findings and research outcomes.

**Figure 2:**
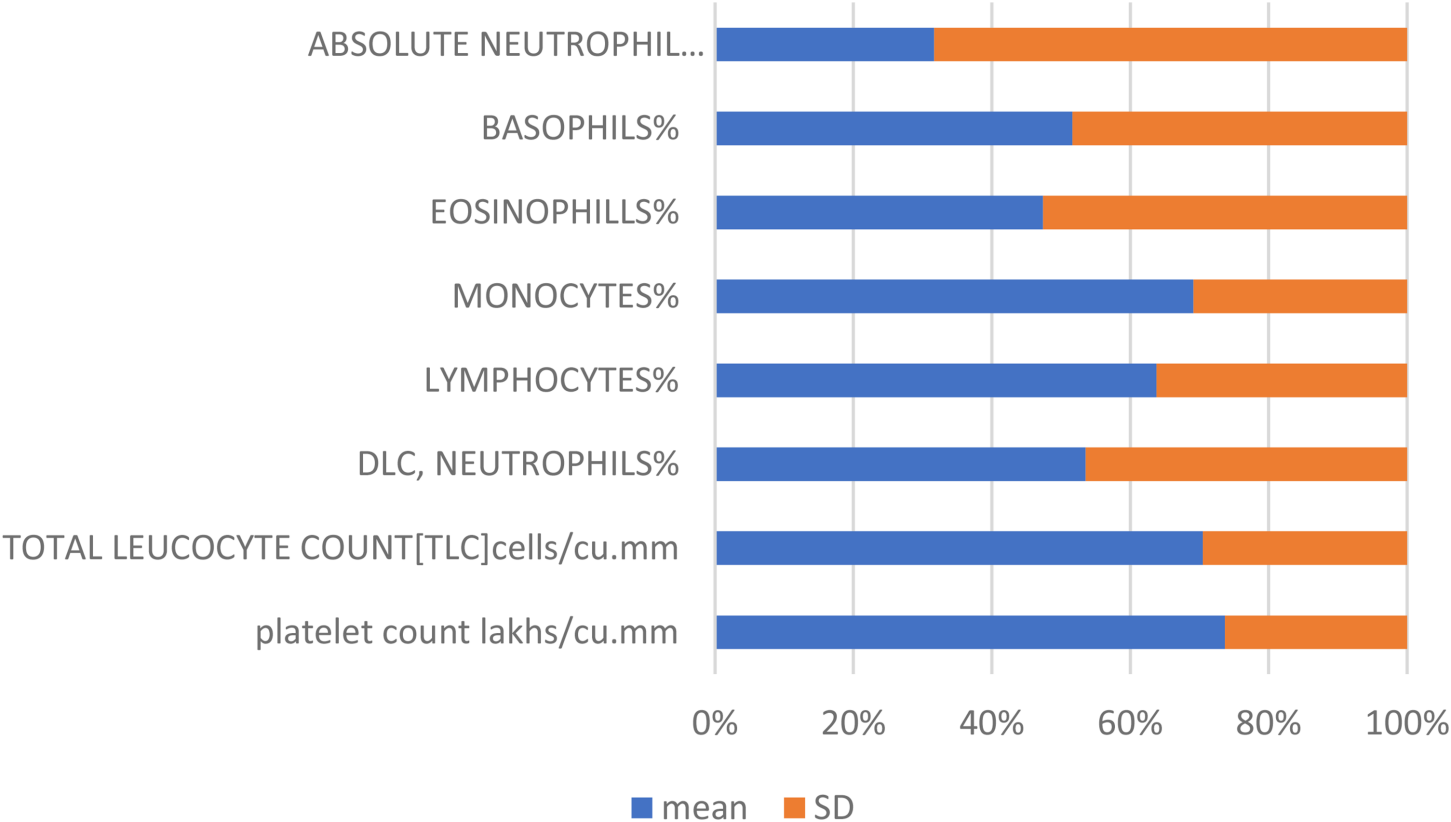
Mean and standard deviation (SD) of biochemical parameters. This figure presents the average values (mean) and variability (standard deviation) of key biochemical parameters, including platelet count, neutrophils, lymphocytes, monocytes, and eosinophils. The mean values provide a measure of central tendency for each parameter, while the standard deviations indicate the extent of variation or dispersion from the mean, offering insights into the consistency and reliability of the data.

Figure 2 and Table 1 provide a detailed summary of the mean and standard deviation for key biochemical parameters, including platelet count, neutrophils, lymphocytes, monocytes, and eosinophils. These percentages reflect the proportional representation of these immune cells within the dataset, offering insights into their relative contributions and distributions.

**Figure 3:**
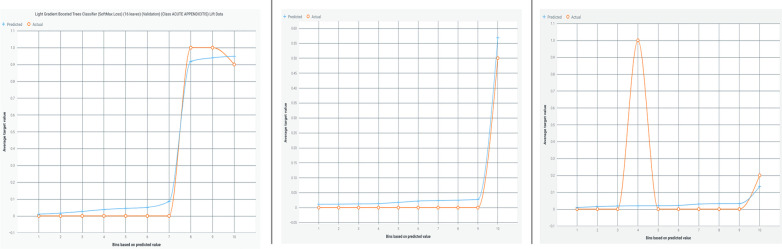
Lift chart for the light gradient boosted tree classifier. This chart illustrates the performance of the Light Gradient Boosted Tree classifier in predicting appendicitis with periodontitis. A lift chart shows the model's effectiveness compared to a baseline model (typically random selection) by plotting the cumulative response of the classifier against the cumulative proportion of positive instances identified. A steeper lift curve indicates that the model achieves higher predictive performance, while deviations from the baseline show its incremental predictive power. This visualization helps assess the model's capability in distinguishing between positive and negative cases, crucial for evaluating its practical utility in clinical and research applications.

Figure 3 illustrates a lift chart for the Light Gradient Boosted Tree classifier, demonstrating a good model fit with both training and test data, and showing minimal overfitting.

**Figure 4:**
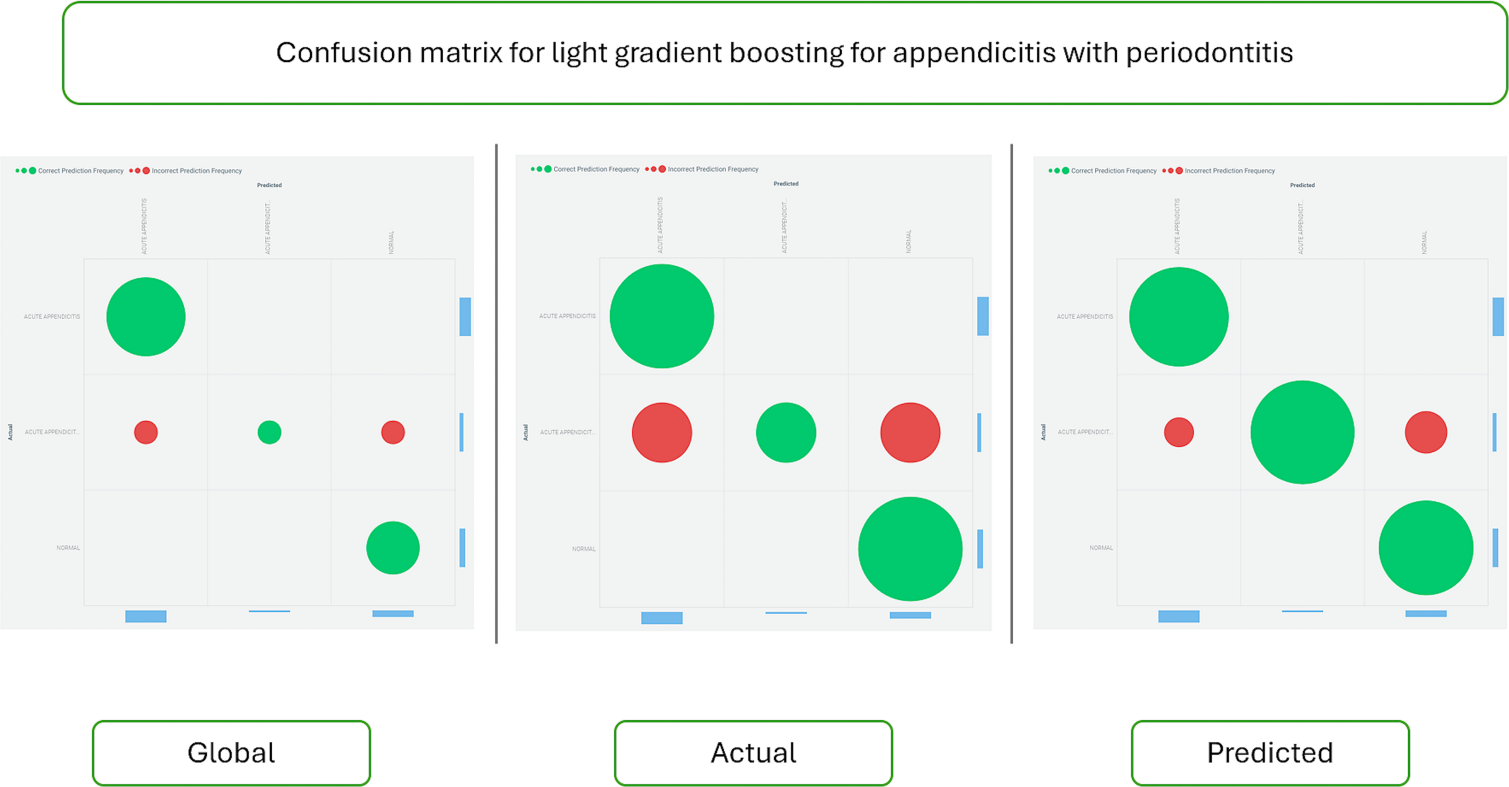
Confusion matrix for the light gradient boost algorithm. This matrix visually represents the performance metrics of the Light Gradient Boost algorithm in predicting appendicitis with periodontitis. The confusion matrix tabulates the true positive (TP), true negative (TN), false positive (FP), and false negative (FN) classifications made by the model. Each cell in the matrix quantifies the number of predictions for each category, enabling assessment of the algorithm's accuracy, sensitivity, specificity, and overall performance. This graphical representation is essential for understanding the model's strengths and weaknesses in correctly identifying cases of appendicitis with periodontitis, providing valuable insights for clinical decision-making and further model refinement.

Figure 4 displays the confusion matrix for the Light Gradient Boost algorithm, providing insight into the accuracy and error rates of the predictions.

**Figure 5:**
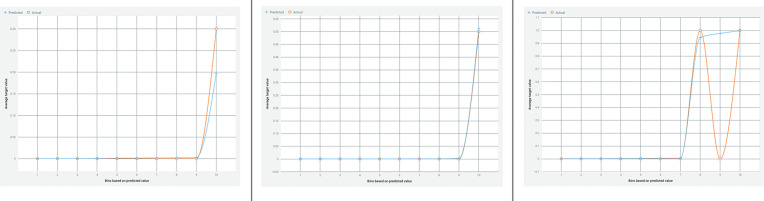
Lift chart for the random forest-based adaBoost classifier. This chart depicts the performance evaluation of the Random Forest-based AdaBoost classifier in predicting appendicitis with periodontitis. A lift chart visualizes the model's effectiveness by plotting the cumulative response against the cumulative proportion of positive instances identified. It compares the classifier's predictive power to a baseline model, typically random selection, showing how much better the model performs. The lift curve's shape indicates the model's ability to distinguish between positive and negative cases, with a steeper curve indicating higher predictive accuracy. This graphical representation aids in assessing the classifier's utility in clinical and research settings, providing insights into its performance and reliability.

Figure 5 presents a lift chart for the Random Forest-based AdaBoost classifier, which also shows a good model fit with training and test data, and minimal overfitting.

**Figure 6:**
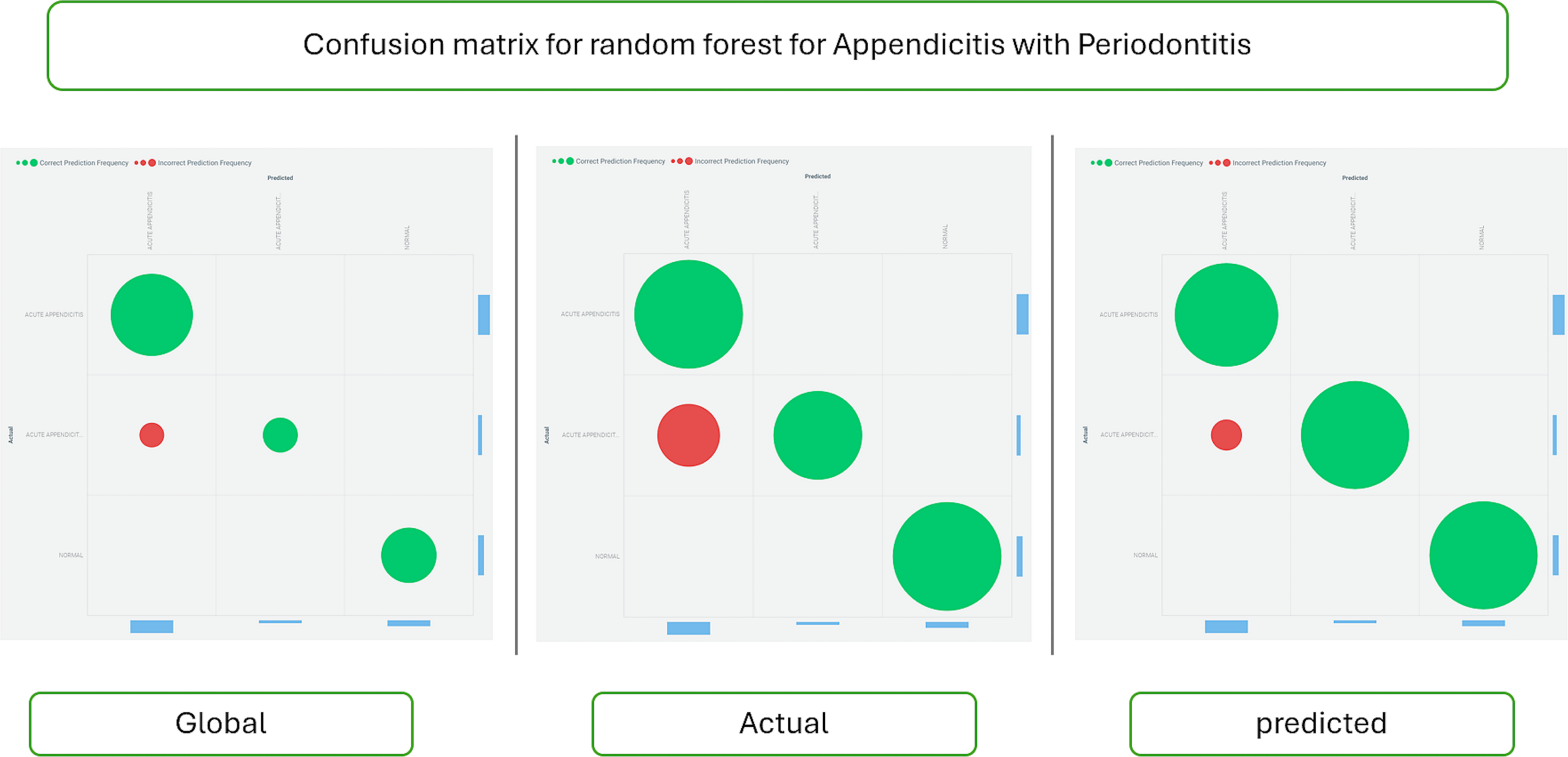
Confusion matrix for the random forest classifier. This matrix visually summarizes the performance metrics of the Random Forest classifier in predicting appendicitis with periodontitis. The confusion matrix tabulates the counts of true positive (TP), true negative (TN), false positive (FP), and false negative (FN) predictions made by the model. Each cell in the matrix quantifies the model's accuracy in classifying instances of appendicitis with periodontitis, providing insights into its sensitivity, specificity, and overall predictive performance. This graphical representation is crucial for evaluating the classifier's effectiveness in clinical and research contexts, guiding further refinement and application of predictive models.

Figure 6 shows the confusion matrix for the Random Forest classifier, comparing actual and predicted data to assess the model's performance.

In terms of accuracy, the results demonstrate notable differences between the Random Forest and Light Gradient Boost algorithms. The Random Forest model achieved a commendable accuracy of 94%, indicating its robust performance in predicting appendicitis with periodontitis. However, the Light Gradient Boost algorithms surpassed this with an impressive accuracy of 98%. This substantial difference highlights the superior predictive capability and efficiency of the Light Gradient Boost approach in this study. The higher accuracy suggests that the Light Gradient Boost algorithms effectively minimized prediction errors and enhanced the model's ability to distinguish between cases of appendicitis with periodontitis and healthy individuals. This finding underscores the importance of algorithm selection and optimization in developing reliable predictive models for clinical and research applications, emphasizing the potential for enhanced diagnostic accuracy and patient care outcomes.

## Discussion

Appendicitis (21, 22), a common cause of acute abdominal pain, continues to present challenges in determining its definitive etiology, as traditional beliefs about luminal blockage being the primary cause are questioned. Recent research suggests that blockage may be more of a consequence than a cause of the condition. Despite fecaliths being frequently associated with appendicitis, their presence is not ubiquitous. Intraoperative pressure measurements typically remain within normal ranges until the disease progresses to advanced stages. The presence of lymphoid follicles in the appendix's submucosa indicates that acute inflammation could be the underlying trigger. It is hypothesized that infections causing luminal obstruction lead to lymphoid hyperplasia, thereby precipitating appendicitis. This theory aligns with the age distribution of appendicitis, which peaks during the period of highest lymphoid tissue activity, typically between ages 10 and 30 (5, 23, 24).

Several studies have implicated oral microorganisms, particularly *Fusobacterium* (3, 25, 26), in the pathogenesis of appendicitis. The prevalence of these bacteria in appendectomy specimens and their correlation with the severity of appendicitis suggest a potential role in mucosal and submucosal inflammation. Furthermore, indicators of oral health, such as the OHI-S and DMFT index values, were significantly elevated in cases undergoing appendectomy, indicating a possible association between poor oral hygiene and increased risk of appendicitis. Dietary factors (22) emphasizing low-fiber diets and unhygienic environments further underscore the potential interplay between oral health and the incidence of appendicitis. This study incorporates biochemical data and clinical parameters such as probing depth, with mean and standard deviation illustrating favorable data distribution (Figures 1, 2; Table 1). Our investigation utilizes advanced algorithms for predicting appendicitis with concurrent periodontal disease, aiming to enhance diagnostic precision and therapeutic strategies in clinical practice.

Light Gradient Boosting Tree (18, 27) represents a rapid and efficient machine learning methodology known for its speed and effectiveness. It leverages histograms to accelerate tree construction, making it particularly suitable for handling large datasets. LightGBM excels in tasks requiring leaf-wise tree growth and gradient-based one-side sampling. Contrasting it with random forests, which train trees independently, gradient-boosting trees sequentially correct errors from prior trees. This sequential nature means random forests can train trees in parallel, while gradient-boosting trees require sequential training. Random forest trees can produce outputs in any sequence due to their independence, whereas gradient-boosting trees adhere to a predetermined order that cannot be altered (28).

Previous research indicates that the Shera score effectively identifies low-risk children suitable for early discharge in acute appendicitis cases (29). However, it does not directly identify candidates for surgery. Children classified as medium to high risk should undergo preoperative ultrasound, MRI, or low-dose CT if uncertainties persist. Among 382 patients studied, 63.9% presented with complicated appendicitis. Age, white blood cell count, and symptom duration emerged as independent factors in children under five. A prediction model validated with an AUC of 0.830 outperformed PAS and ALVARADO scores, underscoring its effectiveness in identifying complicated appendicitis (1) and supporting timely clinical decision-making.

On the other hand, although intra-abdominal abscess (IAA) formation is a significant postoperative complication after appendectomy, recent studies suggest that the surgical technique (open vs. laparoscopic appendectomy) does not significantly affect the incidence of IAA. A retrospective study comparing IAA formation after open and laparoscopic appendectomy found no significant differences in IAA incidence between the two groups (3.73% vs. 3.41%, respectively). However, laparoscopic appendectomy offers advantages such as shorter hospital stay and earlier return to activities (30). Additionally, it is essential to note that acute appendicitis in immunosuppressed patients poses a diagnostic challenge and is associated with increased morbidity and mortality. The decision to perform appendectomy or expectant management in these patients is particularly complex for physicians (31).

Feature selection using a weak classifier learning approach will aid in identifying and predicting the appendicitis with periodontal disease class. The Random Forest model's accuracy (Figures 3–6) is 94%, whereas the Light Gradient Boost algorithms achieve 98%, indicating superior performance in this study. The findings suggest that addressing poor oral health (32) may serve as a preventive measure against appendicitis, highlighting avenues for further investigation into this novel association despite limitations such as a small sample size.

While our study demonstrates promising results in predicting appendicitis in patients with periodontal disease using advanced machine learning algorithms, it's important to acknowledge the limitations that may impact the generalizability of these findings to a broader patient population. Our study was conducted using data from 125 patient records at a single institution, which may not fully represent the diverse demographics and clinical characteristics found in larger, more varied populations. Consequently, the model's performance might differ when applied to populations with different genetic backgrounds, environmental exposures, or healthcare access. Additionally, while the Light Gradient Boost algorithm showed superior performance in our study, the generalizability of this model may also depend on the quality and type of data available in different healthcare settings. Variations in data collection methods, clinical practices, and patient management could affect the model's predictive accuracy. Future research should validate the model across multiple institutions and a more diverse patient population to ensure its robustness and accuracy in various clinical settings.

The absence of follow-up data in our study introduces a potential bias that could affect the accuracy and reliability of our predictive model. Without follow-up information, it's challenging to assess the long-term outcomes of patients who were predicted to have a higher risk of appendicitis in the presence of periodontal disease. This limitation may lead to an incomplete understanding of the model's true predictive performance, particularly in terms of false positives or false negatives that could only be identified through longitudinal patient monitoring. To mitigate this bias, future studies should incorporate longitudinal follow-up to capture a more comprehensive picture of patient outcomes and validate the model's predictive capabilities over time.

The external validity of our findings, or the extent to which these results can be generalized to populations beyond the study sample, is an important consideration. Our study was conducted using patient data from a single institution, which may limit the generalizability of the findings to other settings, particularly those with different patient demographics, healthcare practices, and environmental factors. To enhance the external validity of our findings, future studies should involve multi-center collaborations that include a diverse range of patient populations and healthcare settings. Such studies would help to validate the model's performance across various contexts, ensuring that it remains robust and reliable when applied to different populations. Additionally, external validation with independent datasets is crucial to confirm the model's generalizability and establish its broader clinical utility.

The potential residual effects of previous medication use represent an important factor that could influence the outcomes observed in our study. Medications taken by patients prior to their diagnosis of appendicitis or periodontal disease could have lingering effects that may impact the biochemical and clinical parameters used in our predictive model. Future research should aim to include comprehensive medication histories to assess their impact on predictive modeling. By doing so, it would be possible to adjust for or stratify the effects of prior medications, leading to more accurate and reliable predictions. Additionally, exploring the interactions between different medications and their combined effects on disease biomarkers could provide further insights into optimizing predictive models for clinical use.

In the context of our study, the selection of specific statistical tests was driven by the nature of the data and the research objectives. We employed feature selection methods and machine learning algorithms, such as Random Forest and Light Gradient Boosting, to identify key predictors of appendicitis in patients with periodontal disease. The choice of these algorithms was justified by their ability to handle complex, non-linear relationships within the data and their proven effectiveness in predictive modeling tasks (1, 19, 20). In this study, the primary comparisons were between the performance of the Random Forest and Light Gradient Boosting models, which were central to our research question. To further mitigate the risk of multiple comparison issues, future studies could employ statistical corrections. These methods help control the overall error rate when conducting multiple tests, ensuring that the conclusions drawn from the analysis are more robust and less likely to be influenced by random variation (33).

One significant limitation of our study is its retrospective design. Retrospective studies rely on the analysis of pre-existing data, which may introduce several challenges and potential biases. Furthermore, the retrospective design limits our ability to establish causal relationships between periodontal disease and appendicitis. Despite these limitations, the retrospective design allowed us to leverage existing data to explore the relationship between periodontal disease and appendicitis, providing valuable insights that can inform future prospective studies. Moving forward, prospective studies with controlled data collection and longitudinal follow-up would be essential to confirm and build upon our findings, reducing the biases and limitations associated with retrospective analyses.

## Conclusions

Our study underscores that age, white blood cell count, and symptom duration are critical predictors for identifying concurrent periodontitis in acute appendicitis cases. The developed prediction model represents a novel and promising approach, offering valuable insights into distinguishing between periodontitis and acute appendicitis. These findings suggest significant potential for enhancing diagnostic accuracy and guiding clinical decision-making in cases presenting with both conditions.

## Data Availability

The original contributions presented in the study are included in the article/Supplementary Material, further inquiries can be directed to the corresponding authors.

## References

[1] Celik B Nalcacioglu H Ozcatal M Altuner Torun Y. Role of neutrophil-to-lymphocyte ratio and platelet-to-lymphocyte ratio in identifying complicated appendicitis in the pediatric emergency department. Ulus Travma Acil Cerrahi Derg. (2019) 25(3):222–8. 10.5505/tjtes.2018.06709 31135939

[2] Benedetto G Ferrer Puchol MD Llavata Solaz A. Suspicion of acute appendicitis in adults. The value of ultrasound in our hospital. Radiologia (Panama). (2019) 61(1):51–9. 10.1016/j.rx.2018.08.007 30290969

[3] Gudjonsdottir J Marklund E Hagander L Salö M. Clinical prediction scores for pediatric appendicitis. Eur J Pediatr Surg. (2021) 31(3):252–60. 10.1055/s-0040-1710534 32455443

[4] Zachos K Fouzas S Kolonitsiou F Skiadopoulos S Gkentzi D Karatza A Prediction of complicated appendicitis risk in children. Eur Rev Med Pharmacol Sci. (2021) 25(23):7346–53. 10.26355/eurrev_202112_27428 34919234

[5] Prachanukool T Yuksen C Tienpratarn W Savatmongkorngul S Tangkulpanich P Jenpanitpong C Clinical prediction score for ruptured appendicitis in ED. Emerg Med Int. (2021) 2021:6947952. 10.1155/2021/6947952 33777454 PMC7981174

[6] Bolia R. Diagnosing appendicitis on the basis of clinical prediction rules: are we there yet? Indian J Pediatr. (2023) 90:1173–4. 10.1007/s12098-023-04771-x 37477860

[7] Baima G Marruganti C Sanz M Aimetti M Romandini M. Periodontitis and COVID-19: biological mechanisms and meta-analyses of epidemiological evidence. J Dent Res. (2022) 101(12):1430–40. 10.1177/00220345221104725 35774019

[8] Ma C Wu M Gao J Liu C Xie Y Lv Q Periodontitis and stroke: a Mendelian randomization study. Brain Behav. (2023) 13(2):e2888. 10.1002/brb3.2888 36621868 PMC9927832

[9] Liu J Zhang D Cao Y Zhang H Li J Xu J Screening of crosstalk and pyroptosis-related genes linking periodontitis and osteoporosis based on bioinformatics and machine learning. Front Immunol. (2022) 13:955441. 10.3389/fimmu.2022.955441 35990678 PMC9389017

[10] Yadalam PK Pandian RK Ravishankar PL Vartharajan K Subramaniam S Dinakar M. Evaluation of anticardiolipin antibodies in tobacco users and non-tobacco users with severe chronic periodontal disease. J Int Soc Prev Community Dent. (2016) 6(3):256–60. 10.4103/2231-0762.183115 27382544 PMC4916802

[11] Rangaiah Mahalakshmi M Leela RP Yadalam PK Rajula PB Vadivelu SA Maharshi Malakar V. Estimation of red-complex bacteria in diode laser treated chronic periodontitis patients: a clinical and microbiological study. J Pharm Bioallied Sci. (2020) 12(5):S140–5. 10.4103/jpbs.JPBS_45_20 33149445 PMC7595557

[12] Yadalam PK Krishnamurthi I Srimathi R Alzahrani KJ Mugri MH Sayed M Gene and protein interaction network analysis in the epithelial-mesenchymal transition of Hertwig's epithelial root sheath reveals periodontal regenerative drug targets—an in silico study. Saudi J Biol Sci. (2022) 29(5):3822–9. 10.1016/j.sjbs.2022.03.007 35844389 PMC9280257

[13] Joseph B Yadalam PK Anegundi RV. Management of oral lesions following COVID-19 vaccination. Oral Dis. (2022) 28(S2):2634–5. 10.1111/odi.14342 35933735 PMC9538565

[14] Tolstunov L Hamrick JFE Broumand V Shilo D Rachmiel A. Bone augmentation techniques for horizontal and vertical alveolar ridge deficiency in oral implantology. Oral Maxillofac Surg Clin North Am. (2019) 31(2):163–91. 10.1016/j.coms.2019.01.005 30947846

[15] Luo S Li W Li Q Zhang M Wang X Wu S Causal effects of gut microbiota on the risk of periodontitis: a two-sample Mendelian randomization study. Front Cell Infect Microbiol. (2023) 13:1160993. 10.3389/fcimb.2023.1160993 37305424 PMC10248501

[16] Karabayir I Goldman SM Pappu S Akbilgic O. Gradient boosting for Parkinson’s disease diagnosis from voice recordings. BMC Med Inform Decis Mak. (2020) 20(1):228. 10.1186/s12911-020-01250-7 32933493 PMC7493334

[17] Zhang H Ge L Zhang G Fan J Li D Xu C. A two-stage intrusion detection method based on light gradient boosting machine and autoencoder. Math Biosci Eng. (2023) 20(4):6966–92. 10.3934/mbe.2023301 37161137

[18] Ali F Kumar H Patil S Kotecha K Banjar A Daud A. Target-DBPPred: an intelligent model for prediction of DNA-binding proteins using discrete wavelet transform based compression and light eXtreme gradient boosting. Comput Biol Med. (2022) 145:105533. 10.1016/j.compbiomed.2022.105533 35447463

[19] Hu X Yin S Zhang X Menon C Fang C Chen Z Blood pressure stratification using photoplethysmography and light gradient boosting machine. Front Physiol. (2023) 14:1072273. 10.3389/fphys.2023.1072273 36891146 PMC9986584

[20] Heo J Yoon JG Park H Kim YD Nam HS Heo JH. Machine learning-based model for prediction of outcomes in acute stroke. Stroke. (2019) 50(5):1263–5. 10.1161/STROKEAHA.118.024293 30890116

[21] Feng H Yu Q Wang J Yuan Y Yu S Wei F Development and validation of a clinical prediction model for complicated appendicitis in the elderly. Front Surg. (2022) 9:905075. 10.3389/fsurg.2022.905075 35756469 PMC9218209

[22] Yang KT Wei JCC Chang R Lin CC Chen HH. Association between appendicitis and incident systemic sclerosis. J Clin Med. (2021) 10(11):2337. 10.3390/jcm10112337 34071779 PMC8199283

[23] Chao WC Lin CH Chen YM Jiang RS Chen HH. Association between tonsillitis and newly diagnosed ankylosing spondylitis: a nationwide, population-based, case-control study. PLoS One. (2019) 14(8):e0220721. 10.1371/journal.pone.0220721 31369625 PMC6675079

[24] van Amstel P Gorter RR van der Lee JH Cense HA Bakx R Heij HA. Ruling out appendicitis in children: can we use clinical prediction rules? J Gastrointest Surg. (2019) 23(10):2027–48. 10.1007/s11605-018-3997-1 30374814 PMC6773677

[25] Hajibandeh S Hajibandeh S Hobbs N Mansour M. Neutrophil-to-lymphocyte ratio predicts acute appendicitis and distinguishes between complicated and uncomplicated appendicitis: a systematic review and meta-analysis. Am J Surg. (2020) 219(1):154–63. 10.1016/j.amjsurg.2019.04.018 31056211

[26] Bhangu A. Evaluation of appendicitis risk prediction models in adults with suspected appendicitis. Br J Surg. (2020) 107(1):73–86. 10.1002/bjs.11440 31797357 PMC6972511

[27] Li K Yao S Zhang Z Cao B Wilson CM Kalos D Efficient gradient boosting for prognostic biomarker discovery. Bioinformatics. (2022) 38(6):1631–8. 10.1093/bioinformatics/btab869 34978570 PMC10060728

[28] Osman MH Mohamed RH Sarhan HM Park EJ Baik SH Lee KY Machine learning model for predicting postoperative survival of patients with colorectal cancer. Cancer Res Treat. (2022) 54(2):517–24. 10.4143/crt.2021.206 34126702 PMC9016295

[29] Ayeni A Mahmood F Mustafa A Mcleish B Kulkarni V Singhal S Predicting the severity of acute appendicitis in children using neutrophil-to-lymphocyte ratio [NLR] and platelet-to-lymphocyte ratio (PLR). Cureus. (2022) 14(8):e28619. 10.7759/cureus.28619 36185898 PMC9523736

[30] Mulita F Plachouri KM Liolis E Kehagias D Kehagias I. Comparison of intra-abdominal abscess formation after laparoscopic and open appendectomy for complicated and uncomplicated appendicitis: a retrospective study. Wideochir Inne Tech Maloinwazyjne. (2021) 16(3):560–5. 10.5114/wiitm.2021.103942 34691306 PMC8512505

[31] Mulita F Oikonomou N Provatidis A Alexopoulos A Maroulis I. *Roseomonas gilardii* in patient with leukemia and acute appendicitis: case report and review. Pan Afr Med J. (2020) 36:283. 10.11604/pamj.2020.36.283.24834 33117477 PMC7572676

[32] Yadalam PK Rengaraj S Mugri MH Sayed M Porwal A Alahmari NM Designing an immunoinformatic vaccine for peri-implantitis using a structural biology approach. Saudi J Biol Sci. (2022) 29(1):622–9. 10.1016/j.sjbs.2021.09.041 35002459 PMC8716954

[33] Oberg AL Mahoney DW. Linear mixed effects models. Methods Mol Biol. (2007) 404:213–34. 10.1007/978-1-59745-530-5_11 18450052

